# Origins, potency, and heterogeneity of skeletal muscle fibro-adipogenic progenitors—time for new definitions

**DOI:** 10.1186/s13395-021-00265-6

**Published:** 2021-07-01

**Authors:** Osvaldo Contreras, Fabio M. V. Rossi, Marine Theret

**Affiliations:** 1 Developmental and Stem Cell Biology Division, Victor Chang Cardiac Research Institute, Darlinghurst, NSW 2010 Australia; 2 St. Vincent’s Clinical School, Faculty of Medicine, UNSW Sydney, Kensington, 2052 Australia; 3 Departamento de Biología Celular y Molecular and Center for Aging and Regeneration (CARE-ChileUC), Facultad de Ciencias Biológicas, Pontificia Universidad Católica de Chile, 8331150 Santiago, Chile; 4 Biomedical Research Centre, Department of Medical Genetics and School of Biomedical Engineering, University of British Columbia, Vancouver, BC V6T 1Z3 Canada

**Keywords:** Mesenchymal stromal/stem cell, Fibro/adipogenic progenitor, Fibroblast, Adipocyte, Regeneration, Single-cell RNAseq

## Abstract

Striated muscle is a highly plastic and regenerative organ that regulates body movement, temperature, and metabolism—all the functions needed for an individual’s health and well-being. The muscle connective tissue’s main components are the extracellular matrix and its resident stromal cells, which continuously reshape it in embryonic development, homeostasis, and regeneration. Fibro-adipogenic progenitors are enigmatic and transformative muscle-resident interstitial cells with mesenchymal stem/stromal cell properties. They act as cellular sentinels and physiological hubs for adult muscle homeostasis and regeneration by shaping the microenvironment by secreting a complex cocktail of extracellular matrix components, diffusible cytokines, ligands, and immune-modulatory factors. Fibro-adipogenic progenitors are the lineage precursors of specialized cells, including activated fibroblasts, adipocytes, and osteogenic cells after injury. Here, we discuss current research gaps, potential druggable developments, and outstanding questions about fibro-adipogenic progenitor origins, potency, and heterogeneity. Finally, we took advantage of recent advances in single-cell technologies combined with lineage tracing to unify the diversity of stromal fibro-adipogenic progenitors. Thus, this compelling review provides new cellular and molecular insights in comprehending the origins, definitions, markers, fate, and plasticity of murine and human fibro-adipogenic progenitors in muscle development, homeostasis, regeneration, and repair.

## Background

In mammals, skeletal muscle represents ~ 30–40% of the total body mass, regulating body temperature, metabolism, and physical activity. Comprising the musculoskeletal system, striated muscles are responsible for voluntary and non-voluntary movements. Skeletal muscles are recognized as highly plastic tissue, illustrated by atrophic or hypertrophic changes when disused or trained. Mammalian adult skeletal muscle has extraordinary regeneration capabilities upon injury, making the organ a perfect model to study regeneration and repair, and investigate the contribution of adult stem and interstitial cells in settings of acute or chronic injury. The muscle connective tissue (MCT) components are the extracellular matrix (ECM) and its stromal cells, which actively produce, maintain, and remodel this dynamic scaffold during development, homeostasis, and after trauma.

Among the several cell types that participate in muscle regeneration, tissue-resident mesenchymal progenitors play a crucial role by providing signaling cues that modulate other muscle-resident cells’ function, and actively remodel the ECM during this process. Fibro-adipogenic progenitors (FAPs) have been identified as platelet-derived growth factor receptor alpha (PDGFRα, also known as PDGFRA) expressing cells [1, 2]. A growing body of evidence shows that PDGFRα+ FAPs provide regenerative cues to control muscle stem cell (MuSC) expansion, fate, and myogenesis after acute damage and aging [1–7]. Furthermore, the ablation of stromal cells by using mice model expressing the diphtheria toxin receptor (DTR) under the control of the fibroblast activation protein alpha promoter (FAPα-DTR) impairs the long-term maintenance of hematopoiesis, muscle mass, and cachexia [8]. To note, FAPα+ cells are found in most tissues such as bone, salivary gland, visceral adipose tissue, skeletal muscle, and pancreas; express CD90, CD140a, and SCA-1; and so are most likely to be mesenchymal progenitors, hence FAPs in skeletal muscle [8]. These findings have been confirmed by the Rando laboratory using a knock-in *PDGFRα*^*CreER*^:*Rosa26*^*DTA*^ mice model [7], and more recently, by Tsuchida’s group using a similar cell ablation strategy [9]. Indeed, genetic ablation of PDGFRα+ lineage cells leads to impaired MuSC expansion and leucocyte infiltration, leading to deficient skeletal muscle regeneration after acute chemical injury and neuromuscular defects and muscle atrophy [7, 9]. In addition, following limb ischemia, proper muscle revascularization and repair are lost after ablating FAPs [10]. Hence, PDGFRα+ FAPs are required for successful muscle regeneration, repair, and maintenance during tissue homeostasis and in pathological states.

**Fig. 1:**
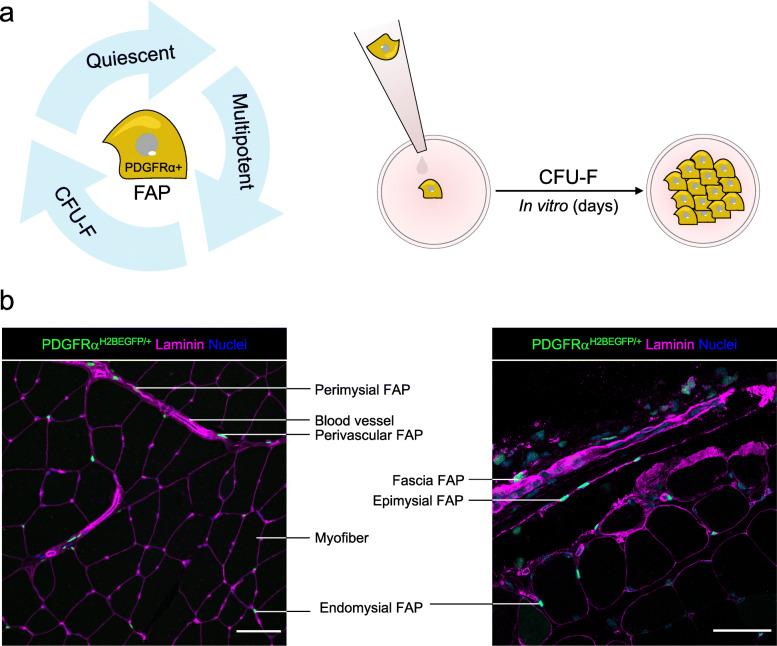
**a** Illustration of FAP cellular properties, including the high expression of PDGFRα, quiescency, CFU-F, and mesenchymal/stromal cell multipotency. Skeletal muscle fibro-adipogenic progenitors form clonal CFU-F following in vitro cell culture. **b** Z-stack confocal images showing the localization of PDGFRα-EGFP^+^ cells in tibialis anterior muscle sections of adult PDGFRαH2BEGFP/+ knock-in mice. Pictures show different skeletal muscle anatomical locations of muscle FAPs. Laminin (magenta) and nuclei (Hoechst, blue) were also stained. Scale bars: 50μm

Muscle-resident PDGFRα+ cells readily initiate fibroblastic colonies (also called fibroblast colony-forming units, CFU-F, (Fig. 1a)) and can clonally differentiate not only into activated fibroblasts/myofibroblasts and adipocytes but also into chondrogenic and osteogenic lineages depending on the context [1, 2, 10–15]. The plasticity and clonal expansion of muscle FAPs are also seen in humans [16]. However, the effects of damage-induced signals and cues on their plasticity, fate, and functions have only recently begun to be explored. The development of new in vivo lineage tracing tools used to identify and track cells expressing specific markers in various animal and damage models in parallel with the recent emergence of single-cell omics have allowed the identification of a broad spectrum of specific stromal populations and their relative contribution to muscle homeostasis, regeneration, and repair.

## The developmental ontology of muscle-resident mesenchymal progenitors

### From the embryo to the adult: role of MCT mesenchymal progenitors on muscle development

Adult MCT is mainly composed of ECM, largely fibrillar collagens type 1 and 3, elastin, fibronectin and proteoglycans, and the supportive matrix-resident stromal cells, also called mesenchymal progenitors or traditionally known as muscle fibroblasts [17–19]. However, compared with the ever-growing knowledge about adult MCT, the composition and the dynamic remodeling of embryonic MCT are poorly understood. While evidence about the ontogeny of interstitial muscle cells exists [20–22], only a paucity of studies have reported their embryonic determination, and hence, the developmental origin and role of these ECM-embedded cells are not yet fully appreciated and understood.

Kardon and colleagues published early evidence of the function of these cells in the formation of limb muscles in the 2000s [23]. The authors described that a mesodermal population of TCF7L2+ cells (formerly known as T-cell factor 4 or TCF-4, a TCF/LEF transcription factor downstream the canonical Wnt/β-catenin signaling) regulates the spatiotemporal determination and differentiation of myogenic progenitors and, therefore, regulates limb muscle development in chicks [23]. Limb TCF7L2+ precursors derive from the lateral plate mesoderm in a muscle-specific pattern, but are different from myogenic precursors since they do not form muscle nor express classical myogenic markers (e.g., *Pax7*) [6, 23, 24]. Thus, myogenic precursors are patterned by extrinsic cues, mostly coming from the MCT, after the cells have migrated through the limb rather than being embryonically predetermined to form particular muscle anatomical structures [23, 25]. These MCT progenitors also influence the myofiber type of limb and diaphragm muscles in a paracrine fashion [24]. Interestingly, not all limb muscles contain TCF7L2+ cells during mouse embryo development, which suggest a distinct patterning and three-dimensional distribution of these cells in different subtypes, or the existence of MCT progenitors that do not express this marker [26]. Nevertheless, TCF7L2 labels a significant proportion of mammalian stromal non-myogenic precursors at birth and during adulthood [24, 27, 28]. Additionally, MuSCs and endothelial cells also express *Tcf7l2* mRNA and protein, albeit at low levels compared with FAPs [7, 24, 28].

Researchers have argued that vertebrate muscles derive from several developmental sources, adding complexity to our understanding of the different origins of MCT in muscle development. For comprehensive reviews, see: [20–22]. Like myogenic precursors, MCT progenitors originate from different and distinct structural origins during embryonic development. In mammals, these include the somites for axial-trunk muscles [29], the lateral plate mesoderm for limb muscles [23, 30], the neural crest cells (NCCs) for head and neck muscles [31–34], and the transient developmental structure originating from the somites called pleuroperitoneal folds (PPFs) for the diaphragm [35]. Remarkably, Merrell and colleagues demonstrated that PPF-resident TCF7L2^+^/GATA4^+^ CT precursors regulate the development of the diaphragm and participate in the etiology of congenital diaphragm hernias (CDH), a type of fibroproliferative developmental disorder [35]. The authors also demonstrated that *Gata4* null mutations in CT progenitors expressing *Paired related homeobox 1 (Prx1)* could cause CDH during diaphragm development. These studies indicate that the aberrant behavior of PPFs CT progenitors can cause congenital muscle diseases like CDH [35].

The studies of Logan’s group have also helped to advance our understanding of the developmental role of MCT precursors in muscle morphogenesis. Initially, through a combination of conditional deletion and advanced imaging techniques, they demonstrated the crucial participation of T-box transcription factors, *Tbx4* and *Tbx5*, in determining the formation of muscles and tendons of the musculoskeletal system [36]. Interestingly, they found that the myoblast-specific loss of *Tbx5* does not affect the correct positioning of myogenic precursors. However, genetic deletion of *Tbx5* and *Tbx4* in the mesenchyme (*paired related homeobox* (*Prx*) expressing lineage), resulted in the perturbation of MCT organization, and therefore, caused mispatterned muscle limbs. Although the authors observed no changes in the expression of *Tcf7l2* in the absence of *Tbx4/5*, the lack of these transcription factors impaired the spatiotemporal distribution of TCF7L2+ cells [36]. Remarkably, the Holt-Oram syndrome, known for leading to skeletal abnormalities and congenital heart disease, is caused by mutations in the *Tbx5* gene [37]. The study of Hasson et al. reinforces the model in which MCT gives rise to muscle pre-patterned structures to guide myogenic precursors during development and further demonstrated that extrinsic MCT-derived cues are critical for muscle morphogenesis. Without surprise, the Transforming Growth Factor beta (TGF-β) signaling pathway is involved in this process. Indeed, Kutchuk et al. demonstrated that embryonic myofibers and C2C12 myoblasts express Lysyl Oxidase (Lox, an enzyme required for cross-linkage formation in elastin and collagen) and that its deletion upregulates the TGF-β signaling. *Lox*^−/−^ mutants display MCT disorganization and delayed myogenesis [38]. Thus, this study illustrates the homeostatic cross-talk between MCT and muscle cells during limb musculoskeletal system development.

The above-proposed model was recently corroborated in detail by Besse and colleagues [39]. These authors took full advantage of an array of labeling and imaging-based studies, mouse genetics, and transcriptomic analyses to establish how individual muscle bundles are generated and established, shedding profound lights on the role of MCT precursors on muscle morphogenesis at unprecedented resolution. They provided a compelling demonstration that muscle morphogenesis is primarily orchestrated by CT mesenchymal progenitors via the secretion of matrix-modifying proteoglycans [39]. Thereby, through the expression and the secretion of a myriad of chemoattractants, ECM components, and growth factors, these stromal cells promote a variety of responses in myogenic precursors, including repulsion, attraction, migration, and patterning [20]. Hence, the MCT creates a developmental pre-pattern that orientates and controls the positioning of myogenic precursors that differentiate into myofibers forming muscle bundles and, consequently, will serve to define the size and shape of muscles, the orientation of its myofibers, and points of origin and insertion on bones [23, 25, 36, 39, 40].

The notion that MCT cells participate in muscle morphogenesis leads to wonder what determines the spatiotemporal dynamics and positional information of MCT precursors. *Hox* genes are a set of genes coding for transcription factors that specify segment identity and provide positional information during animal development [41]. Among them, the caudal *Hox11* genes participate in determining the proximal-distal axis of the musculoskeletal system of limbs [42–45]. *Hoxa11* is broadly expressed through the distal primordium of limb buds at E10.5, but later on, at E14.5, it is exclusively expressed in the CT of tendons, perichondrium, and TCF7L2+ cells, but not in endothelial cells, chondrocytes, osteocytes, nor myogenic precursors [46]. Genetic deletion of *Hoxa11*/*Hoxd11* paralogs, which have a prominent role in patterning bones during development, leads to severe defects in the pattern and regionalization of muscles and tendons, independently of bone defects [46]. Altogether, these results not only demonstrate a previously unappreciated function of *Hox* genes for proper patterning and integration of muscles, tendons, and bones but also illustrated that CT spatiotemporal dynamics participate in the integration of the musculoskeletal system as a whole. Further studies should detail how, when and what factor(s) modulate the spatiotemporal dynamics and positional fate of muscle connective tissue cells.

### Searching for cell-type-specific markers of muscle stromal fibro-adipogenic progenitors

In adult tissue, two studies characterized a population of interstitial muscle-resident progenitors with spontaneous mesenchymal stem/stromal cell (MSC) potential towards fibrous myofibroblast and fatty differentiation [1, 2]. Using fluorescence-activated cell sorting (FACS) of digested mouse skeletal muscle, our laboratory identified and named these cells as fibro-adipogenic progenitors (FAPs) based on their spontaneous differentiation along these lineages [1]. We characterized these progenitors as lineage-negative (Lin−, not expressing hematopoietic (CD45), endothelial (CD31, also known as PECAM-1) or myogenic markers (α7-INTEGRIN) and positive for Stem cell antigen-1 (SCA-1) and CD34 cell-surface antigen expression. Interestingly, while quiescent MuSCs, endothelial cells, and a subset of hematopoietic cells express CD34, its genetic deletion impairs MuSC but not FAP proliferation [47]. We also demonstrated that most Lin-/α7 INTEGRIN-/SCA-1+ cells express high levels of the receptor tyrosine kinase PDGFRα [1]. Similarly, Uezumi et al. characterized the same population using a different gating strategy. They used CD45, CD31, and Sm/C2.6 (MuSC marker) as a negative selection and CD140a (PDGFRα) as positive. They showed that Lin-PDGFRα+ cells express a low level of PDGFRβ and can differentiate in adipocytes, myofibroblasts, and chondrocytes in vitro [2]. They also observed that muscle PDGFRα+ cells were perivascular but did not co-localize with NG2, suggesting that PDGFRα+ cells are not pericytes [2].

PDGFRα+ cells reside in the muscle interstitium and are more abundant in the epimysium and perimysium than in the endomysium. Although most muscle-resident PDGFRα+ progenitors are in close association with blood vessels [1, 2, 48], they are distinct from pericytes. Indeed, pericytes are embedded within the endothelium basement membrane, but PDGFRα+ cells reside outside of vessels. The localization of FAPs is evident around large blood vessels, in which they adopt an adventitial position. With rare exceptions in organs other than muscle, PDGFRα cells do not express defining pericyte markers like *Cspg4* (NG2), *Rgs5*, *Pdgfrβ*, or *Mcam* (CD146) [2, 48, 49]. Notably, while FAPs were initially described in murine muscles, growing evidence indicates that human FAPs have a similar phenotype and functions to mouse FAPs [16, 50–54]. In summary, FAPs (historically called fibroblasts) and the ECM they actively secrete and modify are both significant constituents of the interstitium and perivascular CT.

Distinct subpopulations of CT progenitors exist and express an array of proteins and transcription factors, albeit at variable levels. In the mouse embryo, CT progenitor markers include PDGFRα, TCF7L2, *TBX3/4/5*, *HOX11*, and the Odd-skipped transcription factors OSR1 and OSR2 [21, 23, 26, 40, 55, 56] (Table 1). In murine adult muscles, the large majority of CT fibro-adipogenic progenitors express PDGFRα, SCA-1 (also known as Ly6A/E), CD90 (THY1), CD34, TCF7L2, HIC1, VIMENTIN, DECORIN, and ADAM12 but few of these markers are specific and unique for this heterogeneous population of cells (discussed below) [1, 11, 12, 28, 57, 59, 61, 63, 66] (Table 1). Of note, murine adult muscle PDGFRα+ FAPs express low levels of *Osr1*, which increases upon acute injury in a small subset of FAPs, suggesting the participation of regulatory mechanisms that tightly turn on the expression of *Osr1* resembling developmental-like programs [67]. Remarkably, damage-activated OSR1+ FAPs proliferate faster compared with OSR1- FAPs [67], suggesting that either OSR1 modulates the expansion and functions of FAPs, or it represents an activation marker whose expression increases in proliferating cells.

**Table 1:** Summary of endogenous murine skeletal muscle fibro-adipogenic progenitors

Murine cell	Canonical Markers	Alternative markers	Negative markers	Localization	Differentiation potential	Additional comments	References
Embryonic-fetal FAPs	PDGFRα TCF7L2/TCF4 Osr1	Osr2 Hox11 Tbx3 Tbx4 Tbx5 Sca-1^a^ CD34^a^ Adam-12 Tie-2^a^	CD45 CD31 Ter119 α7-Integrin	Muscle-associated connective tissue and muscle interstitium	Robust in vitro adipogenic and fibrogenic differentiation but low chondrogenic and no detectable osteogenic or myogenic potential. Osr1+ progenitors also give rise to embryonic fibroblast-like cells in the dermis and FABP4+ adipocytes in white fat pads	Little is known about their origin, fate, gene regulation, function, stemness, and self-renewal	[24]; [26]; [57];
Adult FAPs	PDGFRα SCA-1	Hic1 CD90 Decorin (Dcn) PDGFRβ^b^ Col1a1^b^ TCF7L2/TCF4^b^ CD34^b^ Adam-12^c^ Tie-2^c^ Gli1^d^	CD45 CD31 Ter119 α7-Integrin NG2/Cspg4 Rsg5	Fascia, epimysium, perimysium, and endomysium; abundant as perivascular cells	Adipocytes, myofibroblasts, osteocytes, and chondrocytes after muscle injury and in vitro, with no myogenic potential	Required for adult skeletal muscle regeneration and homeostasis; cellular and molecular dysfunction in pathology and disease	[11, 12, 27, 28]; [1]; [13]; [58]; [14]; [58]; [59]; [60]; [61]; [62], [2, 15, 53, 63, 64];

^a^These markers have not been studied in the embryo with detail

^b^These markers are also expressed by different cell types, including satellite cells, pericytes, and endothelial cells

^c^Adam-12 and Tie-2 expression appears to be restricted for a subpopulation of FAPs

^d^Gli1 defines a subpopulation of murine muscle FAPs with pro-myogenic and anti-adipogenic functions [65]

Recently, Gli1 (also known as glioma-associated oncogene 1) expression has been shown to label a subpopulation of muscle FAPs with higher clonogenicity and reduced adipogenic differentiation than Gli negative FAPs [65]. Perivascular cells expressing the zinc finger protein Gli1 undergo proliferative expansion and generate myofibroblasts after kidney, lung, liver, and heart injury [68] and heart injury [6], suggesting that Gli1+ cells are likely a FAP subpopulation as recently shown in skeletal muscles [69].

In humans, cell-surface markers like PDGFRα, CD201, CD166, CD105, CD90, CD73, and CD15 identify skeletal muscle FAPs (Table 2) [16, 51, 53, 54, 64, 71, 72]. Remarkably, the expression of SCA-1 defines a particular cluster of stromal cells within the murine FAP population with different potency and properties in vivo and in vitro, both in the skeletal muscle and heart [66, 73]. However, as SCA-1 does not have a human homolog, its use to identify FAPs is limited by the absence of this antigen in humans.

**Table 2:** Summary of endogenous human skeletal muscle fibro-adipogenic progenitors

Human cell	Canonical Markers	Alternative markers	Negative markers	Localization	Differentiation potential	Additional comments	References
Embryonic-fetal FAPs	PDGFRA	DCN FN1 LUM OSR1 POSTN FAP THY1/CD90 VIM NT5E/CD73 COL1A1 COL1A2 COL3A1 PTN OGN FBLN5	PAX3 PAX7	Similar to what is found in mouse development, although not evaluated in detail	Not evaluated but probably similar to what is found in mouse development	No information about their origin, gene regulation, function, and potency	[70]^a^
Adult FAPs	PDGFRA, CD34 (when negative for CD56, CD31 and CD45)	CD201 CD166 CD105 CD90 CD73 CD34 CD15 COL1A1 TCF7L2/TCF4	CD31 CD45 CD56 α7-Integrin NG2/CSPG4 RSG5	Fascia, epimysium, perimysium, and endomysium; abundant as perivascular cells	Adipocytes, myofibroblasts, osteocytes, and chondrocytes in diseased states and in vitro. Lack of myogenic potential	Increased numbers in diverse pathologies	[16]; [51]; [71]; [72]; [52]; [53, 54, 64]

^a^These other alternative markers suggested by Pyle and colleagues are based on scRNAseq data (Xi et al., [70])

Recently, we showed that the majority of cells expressing the protein-coding gene Hypermethylated in Cancer 1 (*Hic1*) correspond to quiescent muscle-resident FAPs in mice [48]. In adult muscles, HIC1+ progenitors reside in the interstitial space and the myotendinous junctions. In addition to FAPs, small subsets of pericytes (SCA-1−, RSG5+ cells) and tenogenic cells (SCA-1−, SCX+ and FMOD+ cells) express HIC1. Therefore, the expression of *Hic1* comprises a larger proportion of mesenchymal stromal progenitors compared with the expression of PDGFRα, which is limited to FAPs [48]. Along with others, we have confirmed that cardiac PDGFRα+ cells also exhibit multilineage properties in vivo and in vitro [11, 12, 73–75]. In the murine heart, HIC1+ progenitors represent a significant proportion of cardiac FAPs [73]. HIC1 (also known as ZBTB29) is a transcription factor involved in quiescence and cell cycle control [76, 77]. Consistent with these roles, the conditional deletion of *Hic1* induces aberrant cell activation and proliferation of FAPs, impairing muscle regeneration following acute damage and leading to spontaneous development of arrhythmogenic cardiomyopathy-like pathology and signs in the mouse heart [48, 73]. Thus, the unrestrained activation of these progenitor cells and the consequent generation of differentiated progeny are potential pathological drivers of disease.

We claim that the heterogeneity of FAP markers makes sense in a context where the upregulation and downregulation of cell-specific makers participate in modulating the commitment of FAPs into a transitional cell state or differentiation process during lineage progression in response to injury or in disease states. Hence, FAP heterogeneity might contribute to restricting or priming the multipotency of PDGFRα+ FAPs. These are important issues to explore in future research.

## Adult muscle connective tissue and PDGFRα+ progenitors

Adult skeletal muscle contains several cell types that work in unison under tightly regulated conditions to maintain homeostasis. Adult mammalian muscle is a remarkable exception to the low regenerative potential of several organs and tissues like the heart. Before we start highlighting the contribution of endogenous PDGFRα+ cells to mammalian skeletal muscle homeostasis, regeneration, and repair, it is worth revisiting the terminology in this area. Muscle regeneration is defined as the specific substitution or replacement of lost tissue, eventually leading to full restoration of muscle strength. This regenerative capacity relies on resident adult unipotent stem cells (also known as satellite cells), which are quiescent but activate to rebuild this tissue upon injury [6, 78–80].

In comparison, skeletal muscle repair aims to safeguard the remaining function of muscle following solutions of continuity after partial loss of tissue due to massive traumas or chronic insults such as repetitive injuries, disease, and aging. Thus, muscle repair often entails replacing lost myofibers with scar tissue, which acts as a bridge between areas still capable of contraction (for review of tissue regeneration and repair see [81–83]. Therefore, while repair restores muscle integrity, regeneration accounts for restoring tissue function. As observed in other mammalian regenerative tissues such as the liver [81, 84], the form and periodicity of damage can impair the ability of the skeletal muscle to return to homeostasis [59, 85, 86]. Therefore, the current established dual role that PDGFRα+ cells play in acute (regenerative) and chronic damage (reparative/degenerative) suggests that the organization of their intracellular signaling network may integrate opposite complementary signals whose relative strength mainly depends on the type, extension, and frequency of the injury. For this review, we redefine the fate and heterogeneity of muscle-resident PDGFRα+ progenitors and explain their multilineage potentials.

### The adult muscle connective tissue

The muscle environment is complex in structure and several heterogeneous cell types co-exist within it to regulate its function and structure. Although skeletal muscles have an intricate network of blood vessels and nervous tissue, most of their mass is comprised of myofibers. In adults, MCT, which accounts for 1–20% of the total dry mass of muscles, surrounds, protects, and interconnects these primary components [19, 87, 88]. The amount of CT varies significantly from one muscle to another, depending on the anatomical location and physiological function of particular muscles [89]. The adult MCT follows the nomenclature of fascia, epimysium, perimysium, and endomysium accordingly to its location and arrangement within the tissue [90, 91] (Fig. 1b). The topological organization of the covering connective tissue from the outside is described as follows: the fascia, the CT outside the epimysium that surrounds and separates the muscles; the epimysium, which surrounds each muscle group, linking them to the tendons at the myotendinous junctions; the perimysium, which consists of collagen-rich structures that surround the fascicles and interconnect with the epimysium; and the endomysium, which represents a modified basement membrane unsheathing individual myofibers and interconnects to the perimysium [19, 91, 92] (Fig. 1b). These four levels of stromal organization describe the interconnected ECM compartments within muscles. Although each compartment is distinguished by its anatomical position, it is difficult to discriminate each of these ECM compartments in terms of their protein and cellular composition. Remarkably, MCT not only determines the macro and microstructures of embryonic and adult muscles but also connects the myofibers to produce and transmit force. As a result, it increases not only the efficiency of force generation but also protects myofibers from excessive stretching, supporting muscle regeneration and cellular mechanosensation [17–19].

### Muscle-resident fibro-adipogenic progenitors: definitions and identity

Historically, the observation of ECM proteins, such as collagens, being produced and deposited in skeletal muscle suggested the existence of a resident collagen-producing cell within the tissue [17, 18, 90]. Later, numerous observations of CT hyperplasia and interstitial proliferation associated with healing scars in skeletal muscle diseases, including congenital muscular dystrophies, immobilized muscles, and neuromuscular disorders (e.g., amyotrophic lateral sclerosis and denervation) radically increased the attention paid to MCT [93–100]. In order to understand MCT development, establishment, and remodeling, it is crucial to consider the stromal cells that participate in these processes. A critical and challenging step towards a complete understanding of MCT biology has been the identification of a heterogeneous population as the primary effector of ECM deposition and remodeling [11, 12, 17, 18, 48, 62]. Increasing evidence suggests that there are distinct subsets of stromal cells located in discrete yet similar anatomical positions during muscle development and into adulthood [20–22]. This stromal cellular diversity and heterogeneity have been an obstacle to attributing the primary role for matrix deposition to a specific subset of stromal cells.

Jackson and colleagues reported the existence of tissue-resident mesenchymal progenitors with multilineage differentiation capabilities in damaged human muscle over a decade ago [101]. Today, thanks to the great effort of many researchers, we know that adult MCT is mainly produced by muscle-resident PDGFRα+ cells with multilineage progenitor properties and a fibroblast-like phenotype, called FAPs. Increasing evidence suggests that these muscle-resident cells are the primary cellular source of regenerative matrix deposition as well as scarring following muscle injury, disease, neuromuscular disorders, or aging [1, 2, 5, 9, 11, 12, 14, 15, 27, 53, 54, 59, 61, 102–105]. Vallecillo-García and colleagues showed that the source of developmental ECM in limb muscles is a heterogeneous population of PDGFRα-expressing progenitors called embryonic FAPs, closely resembling the population of adult stromal cells we have described, along with other groups [1, 2, 6, 23, 24, 26]. These findings led to some confusion in the nomenclature, with some publications distinguishing between FAPs and fibroblasts, some using the term FAPs as better representing their predominant fibrogenic and adipogenic developmental potential, and some remaining faithful to the historical term fibroblast, which are also known for being heterogeneous and plastic cells. Here, we propose that these muscle-resident multipotent progenitors, whether called FAPs or fibroblasts, are the same cells.

From this point on, the term PDGFRα+ FAPs will refer to muscle-resident CT mesenchymal progenitor with multilineage developmental properties. As discussed below, recent advances in single-cell RNA sequencing demonstrated that FAPs comprise multiple sub-populations, some of which could be bona fide differentiated cells with little developmental potential left [16, 48, 62, 106–109]. This may create a problem with nomenclature diversity, speculation, and high cellular heterogeneity within the adult stromal lineage [110]. FAP heterogeneity is also known to increase following injury and disease, which also complicates their classification and nomenclature [48, 62, 66, 108, 111].

The muscle community has historically described interstitial cells with MSC capability (i.e., fibrogenic, adipogenic, chondrogenic, and osteogenic potency). In addition to PDGFRα+ cells, muscle-resident pericytes have also been proposed to be MSCs that have adapted to the specialized functions required by their adjacent vascular niche. However, although PDGFRα+ FAPs behave as and present defined canonical MSC properties, FAPs are different from tissue-resident pericytic “MSCs.” Indeed, pericytes' cell-surface profile is CD34-/CD45- and CD146+ [112, 113]. Remarkably, Bianco and colleagues revisited the MSC origins and differentiation potential using a broad set of human MSC-like cells (HLA class I, CD73, CD90, CD105, and CD146 positive cells). The authors showed that the cell surface phenotype of “MSCs” isolated from bone marrow, skeletal muscle, periosteum, and cord blood, although quite identical, did not reflect these cells’ cell transcriptomic identity, function, and therefore, their differentiation properties. Thus, “MSCs” are separated from each other, as the authors defined it, by a developmental origin factor [113]. Notably, the authors also showed that CD146+ pericytes are not true MSCs in most of the analyzed tissues, with the possible exception of the bone marrow, where they inherently form bone and bone marrow stroma but lack chondrogenic potential in vivo or myogenic in vitro. On the contrary, in skeletal muscle, CD146+ perivascular pericytes are rather inherently myogenic than skeletogenic [113]. Remarkably, skeletal muscle pericytes are a distinct cell type from MuSCs (CD56+/CD146-) and CD34+/CD146+ endothelial cells that possess a latent myogenic gene signature and potential, and hence, muscle pericytes are committed myogenic progenitors [113, 114]. These pivotal studies have challenged the loose and non-specific MSC nomenclature. However, further studies with lineage tracing and clonal assays are needed to deeply understand stromal cell dynamics in development, homeostasis, and injury and, therefore, to finally faithfully unify their markers, nomenclatures, and definitions.

The abundance of collagen, especially the most abundant protein in animals, type I collagen, determines the stiffness of mammalian tissues [115]. Notably, increased production and deposition of type I collagen fibrils are found after muscle damage. Several cell sources have been suggested as producers of collagen proteins. Using a murine model of increased of increased muscle fibrosis, Chapman et al. corroborated that at least three different muscle-resident cell populations express collagen I, among them PDGFRα+ FAPs. However, muscle progenitors (α7-INTEGRIN)+ and SCA-1+ cells also express the mRNA for this fibrillar matrix protein [17, 18]. These results further confirm our idea that muscle FAPs cannot be solely identified using collagen I reporter mice, but as previously suggested, we strongly recommend employing PDGFRα expression. Since most of the work related to FAPs biology refers to models of single, or repeated rounds of injury, we believe that further studies will likely uncover the role of PDGFRα+ cells in atrophy-related pathologies such as aging-related sarcopenia, cachexia, myasthenia gravis, polytrauma, and neuromuscular disorders. Further research is needed to clarify the existence of subtle differences within stromal cells that might have functional impacts and consequences in muscle physiology, not only during maintenance but also in pathological and disease states.

## Multipotency of muscle-resident PDGFRα+ fibro-adipogenic progenitors

**Fig. 2:**
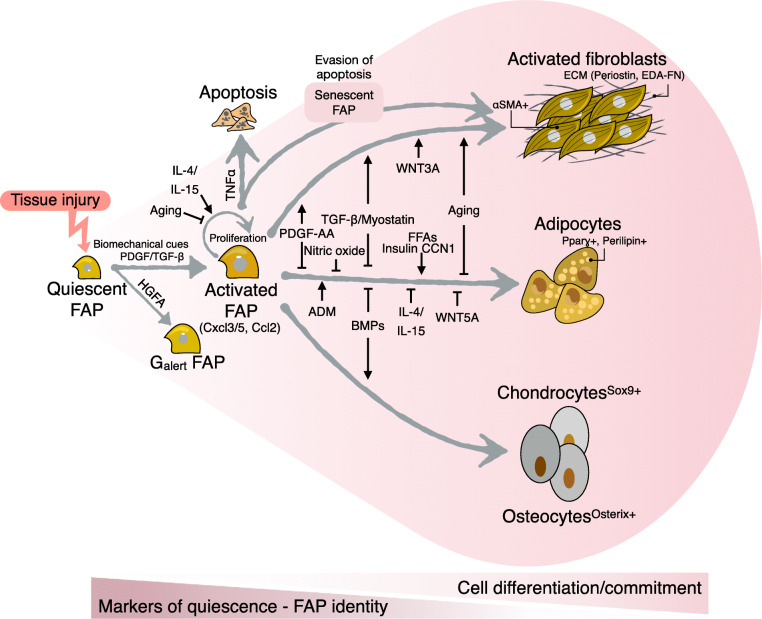
Skeletal muscle FAPs are quiescent cells with multipotency to differentiate towards all the mesenchymal lineages, depending on the degree of activation and tissue damage. Tissue injury and its associated biochemical cues and cell-secreted factors activate muscle FAPs. Activated FAPs act as immunomodulatory stromal cells and signaling hubs before their commitment to more specialized cells. Usually, muscle injury induces the differentiation of them into activated fibroblasts and adipocytes. Severe damage and chronic pathologies tip their differentiation also into chondrogenic and osteogenic lineages. The figure also shows different molecules and factors as well as ligands that regulate their differentiation potential and fate. Notably, many of these molecules hold several steps of FAP life. As quiescent FAPs find their way into activation and cell differentiation, they lose the expression of quiescence markers and their FAP identity but gain cell differentiation markers

In healthy adult muscles, we and others have demonstrated that PDGFRα+ cells represent between ~ 5–15% of the total nuclei and ~ 20–30% of the interstitial mononuclear cells at homeostasis [11, 12, 106, 107, 116]. Stromal PDGFRα+ FAPs display MSC properties and can spontaneously differentiate into adipocytes (rounded, single-vacuole lipid-rich cells, perilipin+ and peroxisome proliferator-activated receptor gamma+ (PPAR*γ*)), activated fibroblasts (long-shaped contractile cells with fibroblast-like morphology, αSMA^+^ (*Acta2*), and highly producing ECM cells), as well as chondrocytes/osteoblasts when bulk cultured, and in clonal assays in vitro and in vivo [1, 2, 11–13,15, 50, 51, 59, 104, 117]. Notably, HGFA, an injury-induced systemic cue, activates muscle FAPs, priming these cells to transition from quiescence into a cellular state with enhanced regenerative potential also known as G alert state [118]. In the following chapters, we discuss FAP multipotency (Fig. 2).

### Fibrogenic potential of PDGFRα+ FAPs

When FAPs are cultured in vitro using standard growth media and 20% oxygen, a large proportion of them will spontaneously differentiate into activated fibroblasts with αSMA^+^ stress fibers [1, 11, 12, 15] (Fig. 2). This demonstrates that FAPs have intrinsic capabilities to differentiate, which is unleashed following their activation and makes *in vitro* studies easily feasible. However, the mechanisms regulating the fibrogenic potential of FAPs remain underexplored.

#### Transforming growth factor-beta signaling

One of the most studied signaling pathways in regulating the behavior and fate of muscle FAPs is the transforming growth factor-beta (TGF-β) signaling pathway. The TGF-β sub-family of cytokines (TGF-β1, TGF-β2, and TGF-β3) are secreted proteins that participate in cell- and tissue-specific biological processes such as wound healing, angiogenesis, immune regulation, apoptosis, tumorigenesis, and proliferation. In pathological conditions, they strongly associate with tissue damage, dysfunction, and fibrosis and are notably mis-expressed (Burks & Cohn, [119, 120]). The complexity of the TGF-β pathway is exemplified by its pleiotropic effects, inducing growth arrest in some cell types but promoting the proliferation of others [121, 122]. TGF-β enhances the proliferation and differentiation of several cell types, including stromal cells (for review, see [123, 124].

When secreted, TGF-β associates non-covalently to a large complex consisting of the latency-associated peptide (LAP) and latent TGF-β-binding protein (LTBP) proteins [125]. Extracellular TGF-β is activated after its release from the LAP-LTBP complex, which can occur via proteolytic rupture or through ECM-cell forces generated by cell traction via the integrin complexes [126–129]. After release, TGF-β binds to its heteromeric serine/threonine kinase type 1 and 2 receptors [TGFBR1/ALK5 and TGFBR2, respectively], and TGFBR3 (also known as betaglycan) co-receptor on the cell surface of the target cell. Of interest, while TGF-β family ligands can bind TGFBR3, this receptor does not have signaling activity on its own, but it modifies the affinity of TGFBR1 and 2 to TGF-β ligands [130]. Indeed, TGFBR3 acts as a co-receptor, amplifying TGF-β signaling activation [131]. TGFBR3 also binds other TGF-β-family ligands such as ACTIVINS, INHIBINS, and bone morphogenetic proteins (BMPs), which we know are primordial proteins for ECM remodeling in skeletal muscle [132, 133]. This co-receptor can also be soluble (by a mechanism called shedding [134]) and could, in some cases, act as an inhibitor of the TGF-β signaling by sequestration of its various ligands [131, 135]. Nevertheless, the function of TGFBR3 in FAP or mesenchymal progenitor behavior has not been studied yet. Its modulation could be a powerful tool as TGFBR3 misexpression is associated with cancer and metastasis [136], in which ECM remodeling is known to be highly active.

Then TGF-β canonical downstream effectors SMAD2 and SMAD3 (R-SMADs) are phosphorylated throughout TGFBR1/ALK5 kinase activity and form a cytoplasmic heteromeric complex with SMAD4 (co-SMAD) [121, 122]. This ternary protein complex translocates to the nucleus where it recognizes SMAD-binding elements (SBE) in the DNA to regulate the expression of diverse target genes [123, 137]. In parallel, SMAD6 and SMAD7 act as inhibitors (also called I-SMADs). Their model of action can be various (via TGFBR1 or SMAD4), but their activation is often a result of a negative feedback loop aiming to downregulate the TGF-β or the BMP signaling pathway [138–140]. TGF-β also activates non-canonical downstream signaling pathways such as ABL, PI3K-AKT, RHO, TAK1, ERK1/2, JNK, and p38-MAPK [124, 141]. In vitro and in vivo experiments suggest that both canonical and non-canonical TGF-β pathways are involved in fibroblast proliferation and myofibroblast differentiation, and thereby modulate TGF-β-induced fibrosis and ECM remodeling [11, 12, 120, 126, 128, 141–143]. However, the specific role of TGF-β canonical and non-canonical pathways in regulating muscle-resident PDGFRα+ FAP plasticity and fate remains underexplored.

In response to muscle injury, TGF-β is produced and secreted by macrophages, FAPs, and regenerating myofibers [11, 12, 59, 144, 145]. Muscle FAPs express the three TGF-β isoforms (TGF-β1, TGF-β2, and TGF-β3) and TGF-β receptors (TGFBR1, TGFBR2, and TGFBR3) [11, 12]. TGF-β ligands through TGFBRs induce FAP-myofibroblast differentiation and ECM production [11, 12, 144, 146]. In addition, TGF-β inhibits the adipogenic priming of muscle FAPs [11], and is pro-mitogenic, and hence, stimulates the proliferation of PDGFRα+ FAPs [11, 12, 59, 144] (Fig. 2). TGF-β signaling pathway activation also seems to be required for FAP survival since the in vivo treatment of mice with SB431542—a selective and potent ALK4, ALK5, and ALK7 receptor inhibitor—reduced the number of expanded FAPs following rotator cuff tear injury [147] (Table 3). Remarkably, we also showed that TGFBR1 and the p38-MAPK protein are responsible for TGF-β-mediated downregulation of PDGFRα [11], associated with a decrease in TCFL2 expression in vitro and in vivo [28]. Thus, as these cells activate, proliferate, and differentiate they lose or reduce the expression of their progenitor state markers (Fig. 2).

**Table 3:** Summary of drug strategies to target muscle fibro-adipogenic progenitor differentiation and fate

Therapy	Target	Cell survival	Proliferation	Cell death/apoptosis	Fibrogenesis	Adipogenesis	References
AG1296	PDGFR kinase activity inhibitor	Not evaluated	Reduced?	Not evaluated	Reduced	Not evaluated	[11]
AICAR	AMPK activator	Reduced	Not evaluated	Induced	Not evaluated	Reduced	Saito et al., 2020 [148]
Azathioprine	Immunosuppressant	Not affected	Reduced	Not affected	Not affected	Reduced	Reggio et al 2019 [149]
Batimastat	MMPs inhibitor (including MMP14)	Not affected	Not affected	Not affected	Not affected	Reduced	[14] [104]
BMS493	Pan-retinoic acid receptor (RAR) inverse agonist	Not evaluated	Reduced	Not evaluated	Reduced	Induced spontaneous differentiation	[69]
Dexamethasone	Glucocorticoid receptor	Induced	Induced	Not affected	Not evaluated	Induced	Dong et al., 2014 [150]
HDAC inhibitors^a^ (TSA and Pracinostat)	HDACs	Not evaluated	Not evaluated	Not evaluated	Reduced	Reduced	[151] [28] [152];
LY2090314 & other GSK inhibitors	GSK3 inhibitors	Slightly decreased	Not affected	Not affected	Mixed results	Reduced	[153]
Metformin	AMPK activator	Not evaluated	Reduced	Not evaluated	Not evaluated	Reduced	[16]
Molsidomine	NO donating molecule	Reduced?	Reduced?	Not evaluated	Reduced	Reduced	[154]
Promethazine hydrochloride	H1 histamine receptor	Not affected	Not affected	Not affected	Not evaluated	Reduced	[72]
SB525334/SB431542	TGFBR kinase activity inhibitor	Reduced	Reduced	Induced after long treatment	Reduced	Not evaluated	[147] [11, 12]
TKIs (imatinib, nilotinib, crenolanib, sorafenib, and masitinib)	Abl, PDGFRs, Kit, DDRs, p38	Reduced	Reduced	Induced	Reduced	Reduced and/or Induced	[59]; [155]; [11, 12]; ^b^[16]; [146];

^a^HDACs-mediated effects on FAP fate are seen only in young mdx but not aged mdx mice

^b^[16] reported that imatinib enhances the amount of perilipin+ FAP-derived adipocytes in vitro

#### Wnt/β-catenin signaling

The Wnt/β-catenin pathway relies on the binding of Wnt ligands to Frizzled receptors and the co-receptors LRP5 and LRP6 at the cell surface to initiate a cascade that regulates the intracellular proteostasis of β-catenin (for recent reviews about the Wnt/β-catenin signaling see [156, 157]. At steady state, the β-catenin pool that is not participating in cell adhesion is bound to a destruction complex, where it becomes phosphorylated and targeted for degradation in a process mediated by the ubiquitin-proteasome system (UPS) [158]. The Wnt ligand-mediated destabilization of the β-catenin destruction complex leads to the accumulation of activated β-catenin (unphosphorylated). Accumulated cytoplasmatic β-catenin subsequently translocates to the nucleus and associates with DNA-binding T-cell factor (TCF) or lymphoid enhancer factor (LEF)–TCF/LEF− transcription factors (TFs) [159]. The binding of β-catenin and TCF/LEF recruits transcriptional partners and chromatin remodeling complexes to regulate the expression of TCF/LEF target genes [160, 161].

Despite the increasing knowledge about the Wnt signaling pathway, the participation of Wnt proteins and signaling in modulating FAP fate has not been investigated until recently. Skeletal muscle SCA-1+ cells (FAPs) are abundant in the muscles of the *mdx* mice (model of the Duchenne Muscular Dystrophy (DMD)), and WNT3a treatment promotes their proliferation and collagen expression both in vitro and in vivo [162]. Interestingly, the treatment of dystrophic mice with DKK1 (Dickkopf 1, a WNT inhibitor) reduced β-catenin protein levels and muscle fibrosis [162]. On the other hand, increased canonical Wnt/β-catenin signaling regulates satellite cell fate and fibrogenic commitment via cross-talk with TGF-β2 in dystrophic *mdx* muscles [163]. Accordingly, we also observed increased β-catenin protein levels upon acute glycerol muscle injury [28]. Xiang and colleagues showed that the conditional genetic loss of β-catenin in heart fibroblast (Transcription factor 21 (TCF21) + cells) and activated fibroblasts and myofibroblasts (Periostin+) lineages reduces fibrosis and ameliorates cardiac hypertrophy induced by pressure overload [164]. In agreement, the sole transgenic overexpression of canonical WNT10B is sufficient to induce fibrosis in vivo [165]. Overall, the Wnt/β-catenin pathway regulates the expression of several ECM genes in fibroblasts from different tissues and organs following injury and disease [28, 164–166].

The outcomes of Wnt/β-catenin signaling depend on the TCF/LEF TFs. However, the potential roles of them in muscle FAPs are underexplored. These TFs recognize TCF/LEF-binding elements and regulatory regions of target genes to regulate gene expression. In this context, we showed the expression of the four Wnt TCF/LEF members in MSC and fibroblast cell lines, as well as tissue-resident FAPs from skeletal muscle and cardiac tissues [28]. We observed that *Tcf7l2* and *Tcf7l1* were the two most highly expressed members, whereas the fibroblast lineage, including FAPs, express *Tcf7* and *Lef1* at lower levels. Moreover, treatment with TGF-β decreases both the mRNA and protein levels of TCF7L2 in PDGFRα+ cells. We described that this regulatory mechanism requires the transcriptional regulation activity of histone deacetylases (HDACs) and the participation of the UPS [28].

Interestingly, TGF-β activates the canonical Wnt/β-catenin cascade and induces nuclear accumulation of β-catenin, which in turn reduced the expression of the WNT inhibitor DKK1 [165]. In agreement with our most recent results showing that TGF-β reduces the expression of several TCF7L2 target genes, whereas it promotes the expression of ECM remodeling genes in idiopathic pulmonary fibrosis and heart fibroblasts [28]. Hence, our work confirms the cross-talk between the Wnt and TGF-β pathways that controls the fate of PDGFRα+ cells and potentially fibrosis (Table 3). In summary, the Wnt cascade modulates TGF-β-mediated effects in fibroblasts, and vice versa [28, 167–169].

#### Platelet-derived growth factor signaling

The platelet-derived growth factor (PDGF) signaling pathway regulates not only vascular development and angiogenesis [170] but also plays crucial roles during development, stem cell fate, migration, and proliferation. PDGF receptors (PDGFRs) are the cell membrane-bound tyrosine kinase receptors for PDGF ligands [171–174]. PDGFs were initially described as serum-derived mitogens essential for fibroblast and smooth muscle cell growth [175, 176]. PDGFs ligands are four gene products consisting of five dimeric isoforms: the homodimers PDGF-AA, PDGF-BB, PDGF-CC, PDGF-DD, and the PDGF-AB heterodimer [177]. PDGFs are known for being released from α-granules of platelets and are potent chemoattractants and mitogens for cells of mesenchymal origin [178]. However, several other cell types express and secrete these ligands, such as inflammatory cells (e.g., macrophages) and fibroblasts [179]. Post-translational proteolytic processing of PDGFs is necessary for their activation. It occurs extracellularly for PDGF-C and PDGF-D but intracellularly for PDGF-A, PDGF-B, and PDGF-AB [178, 179]. A biologically active PDGF ligand is a dimer of two single PDGF chains, which binds one PDGFR.

PDGFRs genes (*PDGFRA* and *PDGFRB*) encode single-pass transmembrane receptors with an extracellular portion of five immunoglobulin-like domains, a transmembrane segment, a juxtamembrane segment, a tyrosine kinase domain, and a carboxy-terminal tail [180]. PDGFRs are monomeric before exposure to PDGF [181]. Its ligand binding-induced dimerization causes their activation, and therefore, later PDGFR de-repression and activation of the receptor's tyrosine kinase activity [180, 182, 183]. Three known functional dimer forms of the receptors exist. They consist of the PDGFRα/α and PDGFRβ/β homodimers and the PDGFRα/β heterodimer [179, 180]. PDGF-AA, PDGF-AB, PDGF-BB, and PDGF-CC promote PDGFRα/α homodimer formation, PDGF-BB, PDGF-CC, PDGF-DD, and PDGF-AB promote PDGFRα/β heterodimer assembly. PDGFRβ/β homodimer can only be induced by PDGF-BB and PDGF-DD isoforms [177, 178, 180]. Although the precise role of PDGF and its receptors in vivo in muscle-resident FAPs is unknown, PDGF signaling seems to regulate FAP survival, activation, proliferation, migration, and fate. In this review, we focused on PDGF ligands and PDGFRα in skeletal muscle health and pathophysiology.

Treatment of *ex vivo* FAPs with PDGF-AA and PDGF-BB ligands activates the PDGF cascade inducing FAP activation and proliferation (Fig. 2) [11, 53]. In addition, upregulated expression of ECM genes and activated downstream ERK1/2, PI3K-AKT, and SMAD2/3 signaling pathways is observed in *ex vivo* FAPs in response to PDGF-AA treatment [53, 184]. By utilizing a pharmacological inhibitor of PDGFR signaling, Mendias and colleagues showed that PDGFR signaling modulates muscle ECM remodeling and angiogenesis upon synergist ablation surgery to induce postnatal muscle growth or hypertrophy [185]. In addition, the treatment with PDGF-AA induces the phosphorylation of PDGFRα and the proliferation of PDGFRα+ cells (Fig. 2) [53]. The authors also suggested, using pharmacological inhibitors, that both PI3K-Akt and MEK2-MAPK signaling pathways are necessary for PDGFRα-induced proliferation [53, 54]. However, persistent PDGF ligand exposure and enhanced PDGFRα signaling levels can cause pathological muscle fibrosis [53, 54, 155, 184]. We have recently shown that PDGF-BB treatment activates proliferative and differentiation-related downstream signaling pathways such as PI3K-AKT, ERK1/2, p38-MAPK, and STAT3 in PDGFRα expressing cells [11, 12]. Recently, Farup et al. showed that PDGF-AA treatment increases the expression of collagen type I in FAPs, whereas it reduces their adipogenic differentiation (Fig. 2). Notably, the PDGF-AA-mediated fibrogenic fate of FAPs associates with a metabolic switch that promotes enhanced glucose consumption [16]. Hence, PDGF signaling could regulate the potency and fate of skeletal muscle FAPs (Fig. 2).

In the heart, Asli et al. showed that PDGF-AB treatment promotes colony formation and self-renewal of cardiac fibroblast, whereas the PDGFR inhibitor, AG1296, suppressed these activities [186]. Interestingly, activated PDGFRαH2BEGFP-mid fibroblasts formed at the expense of resting PDGFRαH2BEGFP-high fibroblasts [73, 186]. These results are in agreement with our recent findings where the expression of PDGFRα changes dynamically during muscle regeneration and repair [11]. Moreover, in vivo PDGF-AB treatment of uninjured hearts did not cause fibroblast activation; however, it increased the number of PDGFRαH2BEGFP-mid fibroblasts after myocardial infarction [186]. Therefore, PDGF-AB isoform targets tissue-resident fibroblasts by increasing the activated fibroblast pool after injury. Interestingly, genetic loss of *Pdgfra* in the resident cardiac fibroblast lineage (TCF21+ cells) results in an overall reduction in the fibroblast population in adult hearts, demonstrating that PDGFRα regulates fibroblast maintenance and homeostasis [187]. Consistently, lineage-specific deletion of *Pdgfra* in *tubulin polymerization-promoting protein family member 3* expressing cell population (*Tppp3+* tendon stem cells) caused impaired tendon regeneration, and therefore, corroborates the cell requirements of PDGFRα signaling for proper tendon healing [188]. Remarkably, the passaging of plastic adherent FAPs obtained from muscles reduces the protein levels of PDGFRα, which associates with their differentiation [12]. Thus, cellular PDGFRα bioavailability may be a modulating factor in PDGF-mediated responses of FAP lineage during survival, fate decisions, and damage-associated behaviors.

### Adipogenic potential of PDGFRα+ FAP cells

Infiltration and deposition of fatty adipose tissue are hallmarks of several skeletal muscle pathologies. However, the cellular and molecular mechanisms underlying fatty infiltration of muscles have not been extensively investigated compared with the ever-growing research in muscle fibrosis. A better understanding of such a discrete fat compartment between myofibers and fascia, also called intra/intermuscular adipose tissue (IMAT), may allow for the targeting of these adipogenic progenitors to increase muscle regeneration and repair.

The lack of reliable cell-specific markers for fat precursor cells has been the main limitation of studying IMAT. As described above, the studies of Joe et al. and Uezumi et al. helped to clarify many aspects of the muscle adipogenic precursor cells. One major focus of these approaches was determining whether IMAT-associated adipocytes were in vivo derived from pre-existent muscle-resident PDGFRα+ cells, other muscle-resident cells, or circulating cells. The work led by Liu et al. in murine skeletal muscle is a classic example of these efforts. The authors suggested that IMAT derives from a lineage of cells not expressing Pax3 (i.e., non-myogenic). They also showed that the genetic ablation of intramuscular adipogenic progenitors based on *Ap2* (also known as fatty acid-binding protein 4 (FABP4)) expression leads to impaired skeletal muscle regeneration, suggesting for the first time that damage-induced fatty tissue may support efficient regeneration upon acute injury [189]. However, AP2/FABP4 expression is commonly thought to be restricted to committed or differentiated adipocytes than progenitor cells [190], questioning the interpretation of the results. Marinkovic et al. [111] showed that Notch signaling is a pivotal pathway regulating FAP adipogenesis in wild-type cells and that dystrophic FAPs are insensitive to Notch-mediated adipogenic inhibition compared with acute injury-derived FAPs [111]. Hence, these results demonstrate that wild-type and dystrophic muscle PDGFRα+ FAPs are in different functional states, which influences their fate and responsiveness to extracellular cues, as previously suggested [62].

Human PDGFRα+ FAPs exist in healthy and DMD pathological muscles, being bona fide counterparts of the PDGFRα+ cells found in mouse muscles [16, 50, 51, 53, 54, 109]. Remarkably, FACS-isolated human FAPs (CD15+/PDGFRα+/CD56−) differentiate towards fully mature adipocytes, phenocopying the in vitro differentiation kinetic and potential of adipose stromal cells obtained from subcutaneous adipose tissue depots [51]. Moreover, when transplanted into a glycerol-damaged muscle (an injury model that promotes adipogenesis) [191, 192], murine FAPs readily differentiate into adipocytes. In concordance to the in vitro report of Liu and colleagues using mouse muscle samples, Arrighi et al. also showed that FAP-derived adipocytes from human muscle biopsies are white rather than beige/brown fat cells. In contrast, Gorski et al. showed increased expression of UCP1, a brown/beige fat cell marker [193], in muscle as well as in FAP cultures following induction of IMAT by glycerol injection [194]. Throughout the body, white fat cells store energy in large, often single, oily droplets. Obesity causes these white adipose tissue cells to multiply and hypertrophy [195, 196]. On the other hand, brown fat cells are equipped with smaller droplets and large mitochondria concentration, giving the tissue its chestnut hue. Hence, in brown adipose tissue, mitochondria produce heat using these fatty droplets, a process also known as thermogenesis [197]. The role of FAP-derived fat cells, whether brown/beige or white, in skeletal muscle health, regeneration, and disease is unknown.

Perhaps the most serious disadvantage of these studies is that they do not directly address the in vivo adipogenic differentiation potential of adult PDGFRα+ FAPs. The definitive proof that muscle PDGFRα+ cells are the main, if not the only, source of injury-induced adipocytes came from lineage tracing experiments using *Pdgfra*^*CreERT*^:*Rosa26*^*EYFP*^ transgenic mice [14]. The authors demonstrated that seven days after acute intramuscular injury, a large proportion of perilipin+ adipocytes derived from PDGFRα+ FAPs, indicating that PDGFRα expressing progenitors are the major source of damage-induced fat cells in normal muscle regeneration and in muscular dystrophy. Indeed, using similar lineage tracing strategies we have demonstrated that cardiac PDGFRα+ FAPs can cause fibrofatty infiltration within the myocardium in an arrhythmogenic cardiomyopathy mouse model driven by the conditional deletion of the quiescence-associated factor Hic1 in heart FAPs [73].

Intriguingly, PDGFRα+ FAPs are ciliated cells and thus possess primary cilium. Conditional deletion of a gene required for ciliogenesis, *Ift88*, in FAPs impaired the injury-induced formation of adipocytes [14]. Mechanistically, the cilia-dependent modulation of FAP adipogenesis involves the participation of Sonic Hedgehog (SHH) signaling, which is repressed in the absence of cilia. Indeed, constitutive activation of the Shh-pathway via genetic deletion of the repressor *Ptch1* was sufficient to block adipocytes’ generation following injury [14]. Remarkably, elimination of the primary cilium in PDGFRα+ FAPs led to enhanced regeneration of myofibers by reducing fatty degeneration of dystrophic muscles, which was also associated with increased myofiber size. The authors also showed that tissue inhibitor of metalloproteinase 3 (TIMP3), an ECM modifier, inhibits adipocyte formation by muscle PDGFRα+ progenitors. Interestingly, aiming to mimic TIMP3 activity, the authors utilized batimastat and showed that the treatment with this pharmacological inhibitor of metalloproteinases prevented injury-induced adipogenesis in vivo [14] (Table 3).

In a different study, Jaiswal and colleagues showed that the treatment with Batimastat prevented FAP spontaneous adipogenesis and reduced fat in dysferlinopathic muscle of dysferlin-deficient (B6A/J) mice [104]. Hence, the authors suggested that the accumulation and adipogenic differentiation of FAP are critical contributors to limb-girdle muscular dystrophy type 2B. Surprisingly, the authors observed no changes in either FAP accumulation, proliferation, or fibrosis as a result of batimastat treatment (Table 3). Nevertheless, the batimastat’s off-targets on other tissue-resident cells such as MuSCs, myofibers, endothelial cells, pericytes, or infiltrating CD45+ cells have not been evaluated yet. Altogether, these findings suggest novel strategies to combat fatty degeneration of chronically damaged muscles by targeting the adipogenic conversion of PDGFRα expressing FAPs to inhibit the deposition of injury- and disease-induced intramuscular fat.

To date, there is not a single clinically approved drug used to prevent IMAT accumulation in muscle disease. However, significant pre-clinical advances have been made. In vivo treatment of *mdx* mice with molsidomine—a nitric oxide (NO) donating molecule—reduced muscle pathology, IMAT accumulation, and fibrosis [154]. These improvements were at least in part mediated by the inhibition of NO-mediated FAP adipogenesis (Table 3). Hence, altered synthesis of NO, a typical finding in DMD, could contribute to enhanced fat deposition. On the search for adipogenic inhibitors, Uezumi and colleagues found that promethazine hydrochloride inhibits, through binding to the H1 histamine receptor, the in vitro and in vivo formation of ectopic adipocytes derived from PDGFRα+ lineage cells in the muscle [72] (Table 3). Promethazine hydrochloride is a first-generation antagonist of the H1 histamine receptor, and therefore, this family of drugs emerge as attractive novel therapeutics against ectopic fat formation in muscle pathologies.

Histone deacetylation leads to the repression of gene expression, and histone deacetylase inhibitors (HDACi, like trichostatin A) provide an exciting means to treat DMD. HDACi have been used in both pre-clinical and clinical studies to improve muscle regeneration and repair in DMD [151, 198–200]. As HDACi treatment inhibits fibro-fatty differentiation of PDGFRα+ FAPs, it reduces the dystrophic pathology through increasing muscle regeneration [151]. Remarkably, in dystrophic FAPs, an HDAC–myomiR–BAF60 molecular network regulates FAP fate, and old FAPs become resistant to HDACi-induced chromatin remodeling compared with young FAPs [201]. Also, HDACi restore the dystrophic-mediated loss of intercellular communication between PDGFRα+ FAPs and myogenic progenitors required for proper muscle regeneration [151], and as recently suggested through an extracellular vesicle-mediated transfer of miRNAs [200]. Interestingly, aging and DMD disease progression limit HDACi-mediated effects [151], which suggests that aging affects the fate of FAPs, as recently detailed by Lukjanenko and colleagues [5]. Recently, Feeley and colleagues showed that rotator cuff tears enhanced HDAC activity in FAPs and trichostatin A inhibited it. HDAC inhibition prevented FAP-mediated fatty infiltration in supraspinatus muscles. Also, trichostatin A regulates muscle FAP adipogenesis by promoting FAP browning (Table 3) [152].

These studies demonstrated that HDACs-mediated pharmacological intervention might counter DMD progression and chronic muscle injury by increasing regeneration by inhibiting fibro-fatty degeneration while favoring the interplay and communication between FAPs and myogenic progenitors. Recently, we have shown that two well-characterized pan-HDACi reduce TGF-β-induced ECM gene expression and also block TGF-β-mediated downregulation of *Tcf7l2* expression [28]. Mechanistically, histone deacetylase inhibitors modulate TGF-β-mediated changes in the expression of TCF7L2 transcription factor target genes of the Wnt pathway [28]. Further investigations should unravel the mechanism by which HDACs regulate the fate of FAPs and how could this be used to target muscle-associated diseases.

In a recent study, Reggio and colleagues used a large drug library screen with pharmacological approaches to demonstrate that the inhibition of the cytoplasmic signaling protein, glycogen synthase kinase 3 (GSK3), reduces PDGFRα+ FAP adipogenesis in vitro, while also repressing muscle glycerol-induced fatty degeneration [153] (Table 3). GSK3 is composed of 2 isoforms (α and β) and is part of the destruction complex of β-catenin, which we showed earlier to play a modulatory role in FAP fate (see the “Wnt/β signaling” section). Mechanistically, the authors suggested that UPS-targeted β-catenin degradation causes an imbalance in the adipogenic fate of dystrophic *mdx* FAPs. The authors also exploited single-cell data and in silico modeling to show that PDGFRα+ FAPs compose the core of the stromal cells in the muscle cell niche by expressing Wnt components and also for being the primary source of Wnt ligands. FAPs seem to actively communicate with endothelial cells, tenocytes, and MuSCs through the production of Wnt ligands. Among the Wnt ligands, they observed that dystrophic FAPs downregulate *Wnt5a* expression compared with wild-type cells. Moreover, WNT5a treatment reduced FAP-induced adipogenesis in vitro by repressing PPAR*γ* expression throughout the activation of β-catenin, suggesting that the Wnt signaling modulates the adipogenic commitment of FAPs in dystrophic muscles (Fig. 2).

On the other hand, Zhao and colleagues recently described that the supplementation of retinoic acid (RA) enhances the proliferation of FAPs at the expense of inhibiting their adipogenic and fibrogenic differentiation [69]. Additionally, treatment of isolated FAPs with a pan-retinoic acid receptor antagonist, BMS493, blocked the RA-mediated effects. Notably, the authors also showed that RA treatment rescued obesity-impaired skeletal muscle regeneration. These findings showed a FAP-type specific effect of RA signaling that regulates skeletal muscle regeneration and repair by means of preserving their progenitor state. Taken together, these findings suggest a novel potential retinoic acid-based strategy to combat chronic skeletal muscle fibro-fatty degeneration of obese patients.

On the contrary, several factors positively regulate muscle FAP adipogenesis. For instance, the matricellular protein CCN family member 1 (CCN1/CYR61) is elevated in the serum and sarcopenic muscles of a murine model of chronic kidney disease and induces FAP adipogenesis [202]. In vivo treatment of mice with the glucocorticoid dexamethasone enhanced IMAT deposition following acute injury (Dong et al., 2014 [150]). Dexamethasone also induces FAP proliferation while increasing their adipogenesis, possibly involving the reduction of IL-4 expression (Dong et al., 2014 [150]). Remarkably, IL-4 administration reduces dexamethasone-induced FAP-derived adipocyte formation, suggesting a novel therapeutic use of IL-4 to reduce IMAT accumulation due to glucocorticoid use in DMD patients (Fig. 2). Perpetuini et al. showed that the glucocorticoid-related molecules, dexamethasone, and budesonide, inhibited the insulin-induced adipocyte formation from mdx-derived FAPs. However, both drugs have a pro-adipogenic impact when the adipogenic mix contains factors that increase the concentration of cyclic AMP. The authors also showed that, only in anti-adipogenic conditions, budesonide suppresses the expression of Pparg, a master adipogenic regulator, via the glucocorticoid-induced-leucine-zipper (GILZ/TSC22D3), and the glucocorticoid antagonist mifepristone alleviates such inhibitory effect [203] (Table 3). This study may shed light on some of the mechanisms underlying the use of glucocorticoids in DMD patients under this kind of treatment. The use of glucocorticoids to treat DMD patients is so far the most common treatment available to delay muscle necrosis and degeneration up to date [204–206]. Finally, the same group, using a similar chemical library-based approach, identified an immunosuppressant drug, azathioprine, that negatively perturbs the intrinsic adipogenic fate, also via PPAR*γ* repression, of wild type and mdx PDGFRα+ FAPs (Table 3).

On the other hand, we recently showed that TGF-β treatment negatively affects FAP differentiation to adipocytes while inducing FAP-to-myofibroblast commitment (Fig. 2). TGF-β1 impairs basal PDGFRα+ FAP differentiation into the adipogenic lineage, by reducing the steady-state percentage of adipocytes but increasing the number of myofibroblasts [11, 207]. Mechanistically, TGF-β treatment reduces the expression of *Pparγ* and *Adiponectin* in skeletal muscle FAPs [11, 12]. We also showed that the adipogenic differentiation of FAPs represses the expression of PDGFRα [11]. Taken together, these studies demonstrate that IMAT-associated adipocytes can derive from pre-existent muscle-resident fibro-adipogenic progenitors.

### Osteogenic differentiation of PDGFRα+ FAP cells

Muscle PDGFRα+ FAPs have osteogenic potential in vitro [2] and when transplanted can successfully engraft and form calcification-rich structures using an in vivo heterotopic ossification (HO) model [208]. HO is a musculoskeletal disorder distinguished by the pathologic formation of extraskeletal bone in muscle, tendon, ligaments, and fascia [209]. BMP2 promotes intramuscular HO regardless of damage; however, BMP9-induced HO requires skeletal muscle injury [210] (Fig. 2). The authors described that intramuscular HO might involve a population of Lin-SCA-1+ cells—likely FAPs [210]. Moreover, Lin^−^/TIE2+/PDGFRα+ progenitors respond to BMP2-stimulated osteogenic commitment and contribute to HO in mice [211]. Additionally, muscle-derived MSCs contribute to fracture repair in a tumor necrosis factor-alpha (TNFα) dependent manner [212]. The above findings are consistent with a recent study of Goldhamer’s group, where the authors employed and characterized a transgenic mouse model that recapitulates a rare autosomal-dominant disorder called fibrodysplasia ossificans progressiva (FOP), which results from a single activating mutation in *ACVR1*; the type I BMP receptor also known as ACVR1/ALK2. The *Tie2*-driven expression of the mutation *Acvr1* R206H is sufficient to phenocopy the spectrum of HO observed in FOP patients [60]. Moreover, they also showed that intramuscular transplantation of mutant *Acvr1R206H/+* FAPs into immunodeficient mice resulted in the formation of HO in an Activin A-dependent fashion. Overall, these data established TIE2+/SCA-1+/PDGFRα+ FAPs as the predominant cell-of-origin and driver of pathological HO. However, it has been suggested that TIE2 is a nonspecific marker for a subset of PDGFRα+ cells since its expression overlaps with other cell populations like endothelial cells, MuSCs, and subsets of hematopoietic cells [2, 213, 214]. Hence, the precise mechanisms and the populations of cells involved in the formation and remodeling of HO remained unknown until then. We recently took advantage of a novel PDGFRα lineage tracing reporter mouse (*Pdgfrα-CreERT2*-*TdTomato*) to further explore the cellular source of muscle ossification [13]. Using a model of BMP2-stimulated intramuscular HO, we showed that a large proportion (~80%) of differentiated osteogenic cells were TdTomato+ after 21 days of muscle injury. Thus, the cell-source responsible for forming ectopic bone in muscle is a subpopulation of muscle-resident PDGFRα+ progenitors [13]. Overall, these studies demonstrate that FAPs are a significant cellular source of chondrogenic cells and osteogenic cells in severely damaged muscles.

Remarkably, intramuscular calcium deposits serve as a pathohistological feature of DMD [215]. Notably, the degree of osteogenic commitment of FAPs appears to match the model of muscle damage and degeneration/regeneration used. Using the severe D2-*mdx* (DBA/2J-mdx) dystrophic mice, which better recapitulates the human characteristics of DMD myopathology, Mázala et al. demonstrated that PDGFRα+ FAPs accumulate within calcified deposits in degenerative muscles [117]. Also, the in vitro osteogenic differentiation of these cells positively correlates with the degree and extension of muscle degeneration and TGF-β levels, which supports previous studies showing that FAPs vastly expand and accumulate accordingly with the extension of damage, TGF-β levels, and fibrosis [11, 12, 15, 27, 53, 54, 59, 103, 117]. In summary, FAP activity and responses are highly contextual, which suggests that signals emanating from the local niche determine their phenotypic multi-lineage-fate. Why are different muscle groups affected to a different extent in muscular dystrophy or neuromuscular disorders? Although several hypotheses might explain this, including muscle fiber type, muscle fiber innervation, muscle of origin, calcium homeostasis, and muscle activity, we still lack information of the role that FAPs play in these processes.

## Fibro-adipogenic cell diversity: Single-cell omics unveil stromal populations in muscles

**Fig. 3:**
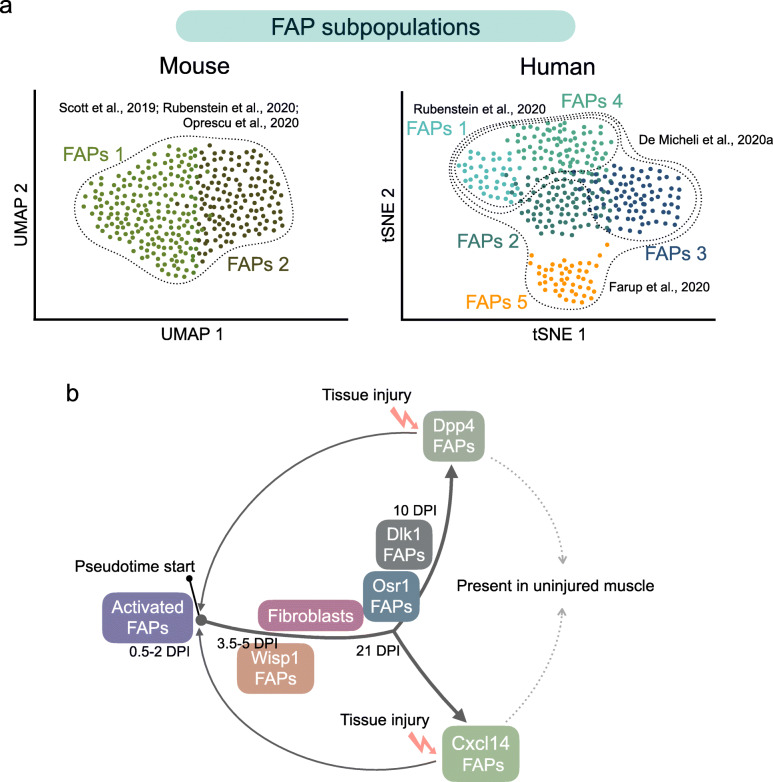
**a** Single-cell RNA sequencing analyses to map muscle-resident FAP mononuclear landscape in murine (left graph) and human (right graph) skeletal muscle tissue. Three different studies, utilizing mice, agree with the existence of at least two principal muscle FAP subpopulations (here shown as FAPs 1 and FAPs 2; see text for details). On the other hand, FAP clustering and FAP subpopulations greatly vary in human muscles. Two different bioinformatic techniques for the presentation of large scRNA-seq datasets and their dimensionality reduction are shown: uniform manifold approximation and projection (UMAP) algorithm and t-Distributed Stochastic Neighbor Embedding (t-SNE). Colored dots represent individual FAP cells. Dotted lines illustrate the different studies discussed in this review. **b** FAP cell trajectories are based on the gene signatures of single cells following damage [48, 108]. The transcriptomes of FAPs indicate high cellular heterogeneity within the FAP populations in response to injury. In mouse muscles, two major FAP subpopulations (Dpp4 FAPs and Cxcl14 FAPs) are present in homeostatic conditions (for detailed markers, see Table 4). Analysis of the pseudotime trajectory of different FAP subpopulations suggests that FAP cells follow a continuum and diverge into two major subclusters upon damage

The recent revolution in single-cell omics technologies, including single-cell RNA sequencing (scRNAseq), single-cell epigenomics (e.i. scATACseq), and single-cell mass cytometry (e.i., CyTOF) has helped to uncover the mysteries of muscle cellular composition and heterogeneity as well as to faithfully recreate a more precise cellular atlas of murine and human adult skeletal muscle in homeostasis, regeneration, and repair [48, 58, 62, 106–109, 111, 216] (Fig. 3). Muscle single-cell analyses faithfully recapitulate key cellular events involved in skeletal muscle regeneration and repair, derived from studies over many years. Such tools and information led us to realize that a complex array of non-myogenic cells (tissue-resident and non-tissue-resident) engage in active cross-talk between each other and with MuSCs to restore tissue function following damage. Single-cell studies evaluate molecular signatures and expression levels of genes or cell surface protein abundance in large numbers of individual cells. They aim to describe at an unprecedented resolution the total interstitial populations of cells in a resting state and to understand their flux in response to injury and disease. Owing to the ability of single-cell omics technologies to refine our understanding of cell heterogeneity by using a plethora of genes and proteins to identify a particular cluster or subpopulation of cells, they are significantly more accurate compared with the use of a single marker to identify cell types.

The classical view of cellular muscle composition is that most of the non-myogenic cells play a positive role and generate a pro-regenerative transitional niche, which, among other functions, support MuSC-driven myogenesis following acute damage [217]. These populations of non-myogenic cells include endothelial cells (CD31+) [218, 219], FAPs (PDGFRα+) [1, 2], connective tissue fibroblasts TCF7L2+ (significantly overlapping with FAPs) [6, 24, 27, 28], pericytes (NG2+, RGS5+) [48], mesoangioblasts [220, 221], tenocytes (TNMD+, SCX+), glial cells (PIP1+, KCNA1+) [48, 58], and a complex array of immune cells [222–224].

With single-cell omics technologies, the transcriptional identity in homeostasis and lineage trajectories of muscle-resident FAPs during the regenerative response and in disease states have started to be discovered. Malecova and colleagues were the first to initially show the existence of cellular heterogeneity in muscle FAPs using single-cell RT-PCR and showed that it increases in regenerating muscles [62] (Table 4). The authors showed that a specific subpopulation of vascular cell adhesion molecule VCAM-1+ FAPs vastly expands and drives muscle fibrosis in acute damaged muscles and adult dystrophic muscles of the mdx mice [62]. Notably, the VCAM-1+ subFAPs are absent in uninjured muscles, suggesting that Vcam1 may be an activation marker. Recently, a population of quiescent HIC1+ mesenchymal progenitor cells has been described. This population contains precursor cells for several mesenchymal lineages including muscle FAPs (*Pdgfra*+, *Ly6a*+), tenocytes (*Tnmd*+), pericytes (*Rgs5*+), and a new subset of cells called myotenocytes (*Col22a1*+) [48]. However, further functional analyses will be required to confirm if the myotenocyte cells present at the regenerated myotendinous junction represent an independent subpopulation of HIC1+ progenitors with a specific function or a differentiated cell state of tenocyte progenitors in the myotendinous niche. Even though HIC1 is a broad stromal progenitor marker, FAP gene signatures segregate from other interstitial populations of mesenchymal cells like pericytes and tenocytes [48]. Remarkably, the gene signature of muscle PDGFRα+ FAPs is heterogeneous and progresses over time after acute muscle injury, which reveals their dynamic role in regeneration [48, 108]. Therefore, FAPs acquire a unique plastic transcriptome that changes as the inflammation progresses and damage resolves through regeneration. Concerning mouse muscles, two major FAP populations have been described (FAP1 and FAP2) in undamaged muscles (Fig. 3a). FAP1 associates with the ECM gene signatures, such as collagens *Col4*, *Col6* and *Col15*, *Lum*, *Sparcl1*, *Podn*, *Smoc2*, *Mgp*, *Cxcl14*, *and Bgn*. On the other hand, the FAP2 subpopulation expresses genes involved in cell signaling and migration, including *Sfrp4*, *Igfbp5*, *Sema3c*, *Dpp4*, *Tgfrb2*, and *Wnt2* [48] (Table 4). In contrast, another study described no segregation of the FAP population, primarily based on the expression of a few FAP markers [216]. These subtle differences might be explained by a number of technical factors such as muscle groups used for scRNAseq, tissue digestion and cell-type enrichment methods, single-cell RNA sequencing platform—although most of them used Chromium 10× Genomics, the number of cells captured and recovered after sequencing, and downstream data processing (e.g., version of Seurat R package used) and interpretation.

**Table 4:** scRNA-seq gene signatures used for FAP identification and clustering in muscle homeostasis

Genes/markers	FAP subpopulations	Species	Reference
*Sca1*, *Cd34*, *Pdgfra*	SubFAPs: Tie2^low^ (Tek), Vcam1^low^	Mouse	[62]
	SubFAPs: Tie2^high^ (Tek), Vcam1^high^		
*Ly6a* (*Sca1*), *Ly6e*, *Pdgfra*, *Dcn*	*Not determined*	Mouse	[58]
*Pdgfra*, *Ly6a* (*Sca1*), *Hic1*	FAP1: *Cxcl14*, *Col4*, *Col6*, *Col15*, *Lum*, *Sparcl1*, *Podn*, *Smoc2*, *Mgp*, and *Bgn*	Mouse	[48]
	FAP2: *Dpp4*, *Sfrp4*, *Igfbp5*, *Sema3c*, *Tgfrb2*, and *Wnt2*		
*Pdgfra*, *Ly6a* (*Sca1*), *Dcn*, *Cd34*	*Not determined*	Mouse	[216]
*Pdgfra*, *Col3a1*, *Dcn*, and *Gsn*	*Not determined*	Mouse	[107]
*Pdgfra*, *Ly6a* (*Sca1*), *Cd34*	FAP1: *Cxcl14*, *Enpp2* (*Autotaxin*), *Crispld2*, *Hsd11b1*, *Smoc2*, *Ccl11*, *Gsn*, and *Dcn*	Mouse	[108]
	FAP2: *Dpp4*, *Pi16*, *Wnt2*, *Igfbp5*, *Igfbp6*, *Fbn1*, and *Ugdh*		
*PDGFRA*, *CD34*, *COLLAGEN 1*, *COLLAGEN 3*, *and COLLAGEN 6*	LUMICAN (LUM) FAP: *LUM*, *DCN*, *CXCL14*, *COLLAGEN 4*, and *COLLAGEN 15*, *SMOC2*, and *GSN*	Mouse and human	[109]
	FIBRILLIN 1 (FBN1) FAP: *FNB1*, *MFAP5*, *LOXL1*, PRG4, *ELN*, *IGFBP5*, and *FSTL1*		
*PDGFRA*	FAP1 (fibroblasts 1): *COL1A1*, *SFRP4*, *SERPINE1*, and *CCL2*	Human	[106]
	FAP2 (Fibroblast 2): *FBN1*, *MFAP5*, and *CD55*		
	FAP3 (Fibroblast 3): *SMOC2*, *ADH1B*, and *ABC18*		
*PDGFRA*, *CD34*, *COL1A1*, *COL6A3*, *TCF7L2*	FAP1: *PCOLCE2*, *MFAP5*, *IGFBP6*, *ENNP1*, *CD55*, and *AXL*	Human	[16] ^a^
	FAP2: *LUM*, *MYOC*, *CCL2*, *ADH1B*, *SFRP2*, *CXCL14*, and *MGP*		
	FAP3: *TNXB*, *C3*, *COL15A1*, *SMOC2*, *ABCA8*, *COL6A1*, and *HMCN2*		
	FAP4: *IGF1*, *CRLF1*, *SCN7A*, *ITIH5*, *PTGDS* and *NOV*		
	FAP5: *SEMA3C*, *PRG4*, *DEFB1*, *CCDC80*, *LINC01133*, and *IGFBP5*		

^a^By re-clustering the FAP population, the authors described the existence of 7 different FAP subpopulations in human muscles [16]

Oprescu et al. [108] reported that murine CXCL14+ FAPs (also expressing *Smoc2*, *Ccl11*, *Gsn*, *and Dcn*) and DPP4+ (expressing *Pi16*, *Igfbp5*, *Igfbp6*, *Fbn1*, and *Ugdh*) FAPs represent two different FAP subtypes present in non-injured murine tibialis anterior muscle, suggesting that FAPs could represent two distinct subpopulations of interstitial cells in resting conditions, as it was previously shown by us [48] (Table 4). However, in response to injury, the two populations follow a linear trajectory into a single population of activated FAPs (highly expressing chemokine genes like *Cxcl5*, *Cxcl3*, *Ccl7*, and *Ccl2*) at 0.5 and 2-day post-injury (DPI), then progressing into WISP1+ FAPs at 3.5 and 5 DPI (highly expressing *Postn*, *Csrp2*, *Sfrp2*, *Ptn*, *Cilp*, and *Cthrc1*), followed by DLK1+ FAPs at 10 DPI (expressing *Itm2a*, *B830012L14Rik*, *Meg3*, *Airn*, *Peg3*, *Zim1*, *H19*, and *Igf2*), and finally two FAP subpopulations at day 21 DPI, OSR1+ (*expressing Gsn*, *Ccl1*, *Bmp4*, *Bmp5*, and *Wnt5a*) and fibroblast FAPs (expressing *Col3a1*, *Col1a1*, *Col1a2*, *Col6a3*, and *Meg3*) [108] (Table 4 and Fig. 3b). Notably, the authors showed that a proportion of the OSR1+ FAPs at 21 DPI diverge into the two populations observed in undamaged muscle: DPP4+ FAPs and CXCL14+ FAPs (Fig. 3b). Therefore, the gene expression of single-cell FAPs is highly diverse, representing a continuum state during skeletal muscle regeneration (Fig. 3b). Owing to the high degree of FAPs diversity, we speculate that FAP subpopulations have adapted to play supportive and distinct roles during regeneration. These data also suggest that the transcriptional diversity of PDGFRα+ FAPs at the single-cell level might reflect their differential developmental potential.

In addition to these studies, Rubenstein et al. [109] described two human FAP subpopulations as LUMICAN (LUM)+ FAP and FIBRILLIN 1 (FBN1)+ FAP subtypes (Table 4 and Fig. 3a). Interestingly, both FAP subpopulations showed specific differences in the expression of Collagen types. The authors also validated the existence of these two distinct FAP subtypes by using scRNAseq of mouse quadriceps and diaphragm muscles [109]. ECM gene expression was also consistent among mouse and human muscles, with *COLLAGEN 1*, *COLLAGEN 3*, and *COLLAGEN 6* broadly expressed across FAP subpopulations. In contrast, LUM+ FAPs express *COLLAGEN 4*, and *COLLAGEN 15* predominantly compared with FBN1+ FAPs (Table 4). Interestingly, the authors reported differences in the gene expression of the precursor gene of *TIE2* protein among the two species, which was expressed only in the FBN1+ FAP subtype found in mouse muscle but none of the FAP subtypes found in human muscles. The meaning of these subtle differences in gene expression across mouse and human muscle FAP subtypes should be address in future research.

De Micheli and collegues collected and integrated ~ 22,000 single-cell transcriptomes generating for the first time a consensus cell atlas of human skeletal muscles [106] (Table 4 and Fig. 3a). The authors described three subpopulations of fibroblasts (likely FAPs) in which *COLLAGEN 1*, *SFRP4*, *SERPINE1* and *CCL2* are highly expressed by fibroblast 1; *FBN1+*, *MFAP5*, and *CD55* are expressed by fibroblast 2, whereas fibroblast 3 highly expresses *SMOC2* [106, 107] (Table 4).

Recently, Farup and colleagues described 5 subpopulations of human muscle FAPs (Table 4 and Fig. 3a). However, by sub-setting the FAP population and re-clustering, the number of clusters increased to 7. The authors reported that the expression of *THY1/CD90* is enriched in cluster 4, whereas *PDGFRA* gene expression is broadly distributed among the FAP subpopulations (Table 4). Remarkably, the CD90+ subpopulation of FAPs is associated with increased fibro-fatty infiltration and seems to drive the muscle degeneration found in obese and type-2 diabetes patients [16]. Although the composition of each cellular interstitial compartment changes dramatically after injury and in disease settings in mice, there is no information about how the FAP population behaves following injury or how degenerative diseases alter its activities in humans. Nonetheless, we and others have detected a diverse range of mesenchymal stromal cells including quiescent subsets, which rapidly expand following injury and secrete cytokines modulating inflammation, trophic factors, and regenerative cues to promote skeletal muscle maintenance, MuSC renewal, and regeneration.

In conclusion, further studies should focus on understanding the mechanisms by which FAP cell heterogeneity arises. We aim to understand the lineage restriction of FAPs by gene regulatory networks and epigenetic factors that, in combination with the extrinsic effects of the spatial context could regulate their fate and plasticity within muscles. Although there has been encouraging progress in understanding FAP phenotypic variability and activities, future research should look to translating this knowledge into efficient medical applications.

## Future perspectives

Skeletal muscle requires a complex orchestra of specialized populations of cells to perform its crucial functions. The origin, behavioral activities, lineage potency, and expression of markers associated with stem or progenitor cell states define these specialized cell types. Here, we have focused on the unappreciated role of PDGFRα+ FAPs in muscle biology, health, structure, and regeneration. Apart from the accepted structural part that the connective tissue provides for proper muscle development, the complex cues and matrix that stromal cells produce are essential to sustain myogenesis and support proper muscle morphogenesis. FAPs are implicated in muscle scarring, disease, and pathology. Although substantial progress has been made in understanding FAP behavior, they remain poorly characterized, and the relationships with other stromal cells are not well understood. PDGFRα+ FAPs and their descendant lineages, including activated-fibroblasts/myofibroblasts, adipocytes, chondrogenic and osteogenic cells, modulate muscle regeneration and repair. These plastic cells play broad roles as sentinels, stress sensors, immune regulators, cellular hubs, and paracrine factories, which are still under active research in multiple pathological settings.

As discussed above, lineage tracing technologies combined with single-cell sequencing strategies should bypass the significant limitations that historically prevented us from deconvolving the complexity of stromal cell populations. The diverse fibroblast nomenclature has periodically led to confusing claims in muscle biology and ensuing turmoil in the literature. Thus, resolving the regenerative vs. reparative dichotomy of muscle-resident mesenchymal progenitors, and distinguishing true lineage heterogeneity from the diverse functional states that these cells can dynamically and reversibly acquire remains a high-priority issue for the field. Despite these various uncertainties, in this review, we establish a baseline for the contribution of fibro-adipogenic progenitors to muscle development, homeostasis, regeneration, and repair.

## Conclusions

In this review, we document new insights about the various properties of muscle-resident PDGFRα+ FAPs and discuss the current state of knowledge on their origins and lineage capabilities. Here we propose to define a cell as FAP if they present the following characteristics: 1. Express PDGFRα at the gene and protein level. 2. It is located in the tissue's interstitium and behaves as a perivascular cell but not residing in the blood vessel cavity. 3. It can form colonies *in vitro*. 4. Can differentiate into activated fibroblasts, adipocytes, chondrocytes, and osteocytes in vitro and in vivo.

We illustrated their importance in maintaining proper muscle function and critical role during the onset and establishment of scarring in pathology and disease. Growing evidence shows that PDGFRα+ cells are heterogeneous and act as signaling hubs by providing regenerative cues and integrating these signals in the muscle niche. Thus, FAPs influence other populations of cells within skeletal muscle and vice versa. Ultimately, by understanding and manipulating the complexity and variability of the stromal compartment, specifically the FAP lineage, we aim to develop novel therapeutics to treat several scar-forming pathologies. It remains plausible to foresee a future where clinical leaps could be made based on these cells and where severe muscle injury could be treated without prolonged myodegeneration and muscle malfunctioning.

## Data Availability

All data generated or analyzed during this study are included in this published article.

## Abbreviations

aSMA: Alpha smooth muscle actin
BMP: Bone morphogenetic protein
CFU: Colony-forming unit
CDHs: Congenital diaphragmatic hernias
CT: Connective tissue
DMD: Duchenne muscular dystrophy
DTA: Diphteria toxin A
DTR: Diphteria toxin receptor
ECM: Extracellular matrix
FAPα: Fibroblast activation protein alpha
FAPs: Fibro-adipogenic progenitors
FOP: Fibrodysplasia ossificans progressiva
GSK: Glycogen Synthase Kinase-3
HO: Heterotopic ossification
IL: Interleukin
IMAT: Intermuscular adipose tissue
LGMD: Limb-girdle muscular dystrophy
Lox: Lysyl oxidase
MSCs: Mesenchymal stem cells
MCT: Muscle connective tissue
MuSCs: Muscle stem cells
NCCs: Neural crest cells
PDGFRα: Platelet-derived growth factor receptor alpha
PDGFRβ: Platelet-derived growth factor receptor beta
PDGF: Platelet-derived growth factor
PPFs: Pleuroperitoneal folds
OSR: Odd-skipped-related
Shh: Sonic Hedgehog signaling
Sca-1: Stem cell antigen-1
Tbx: T-box transcription factor
TIMP3: Tissue inhibitor of metalloproteinases 3
TCF21: Transcription factor 21
TGF-β: Transforming growth factor beta
UPS: Ubiquitin-proteasome system
